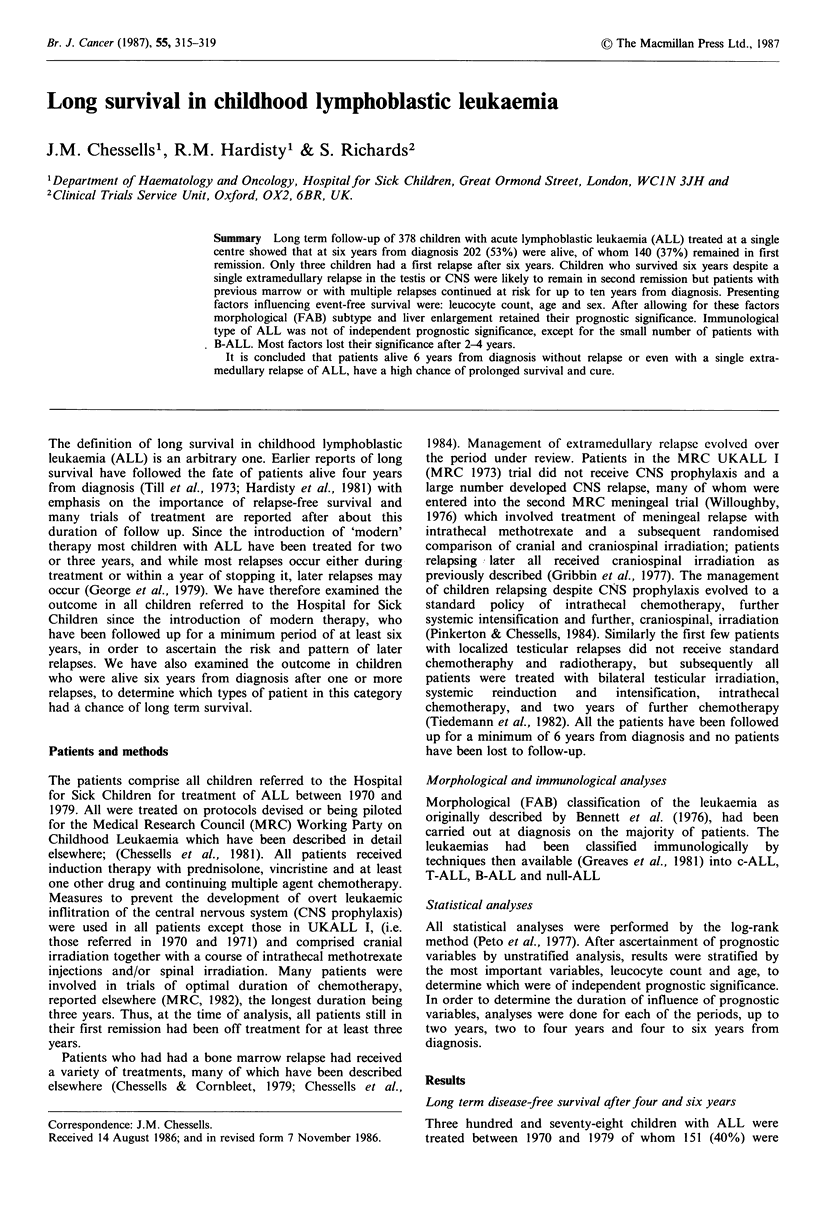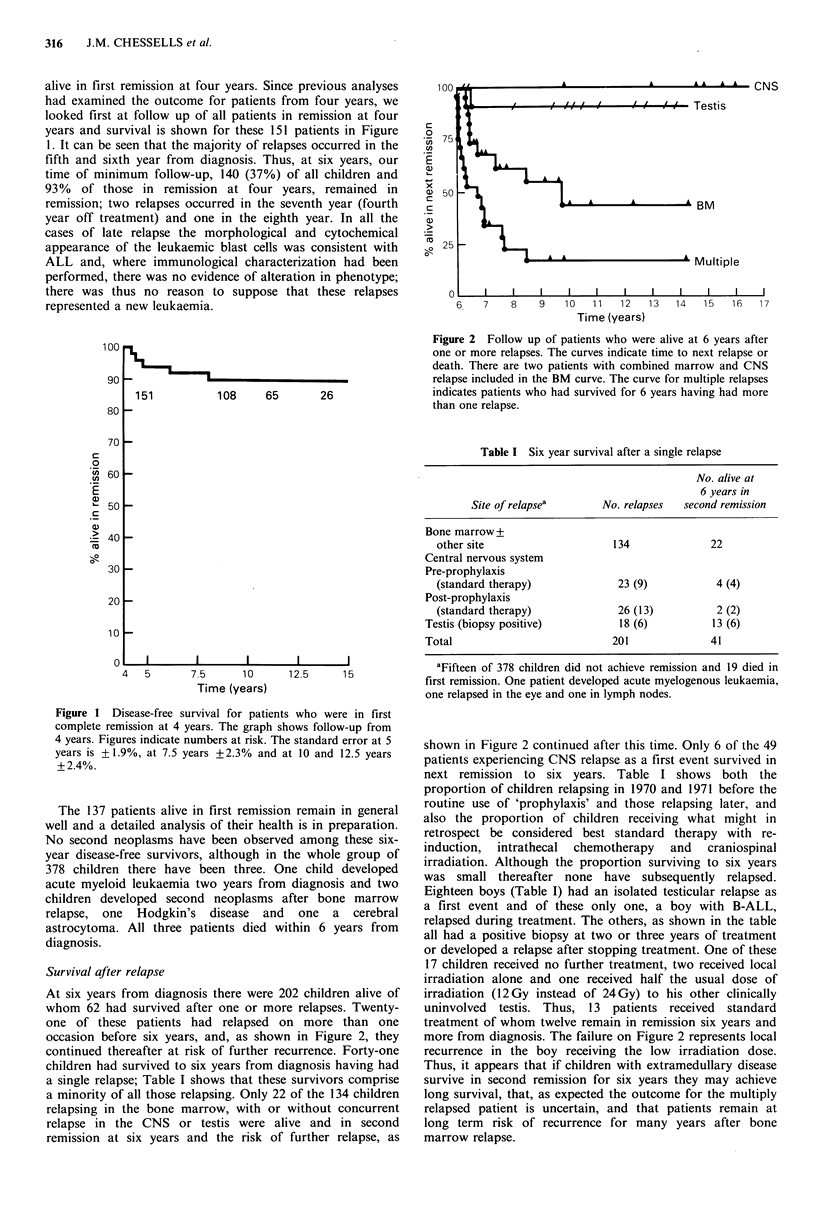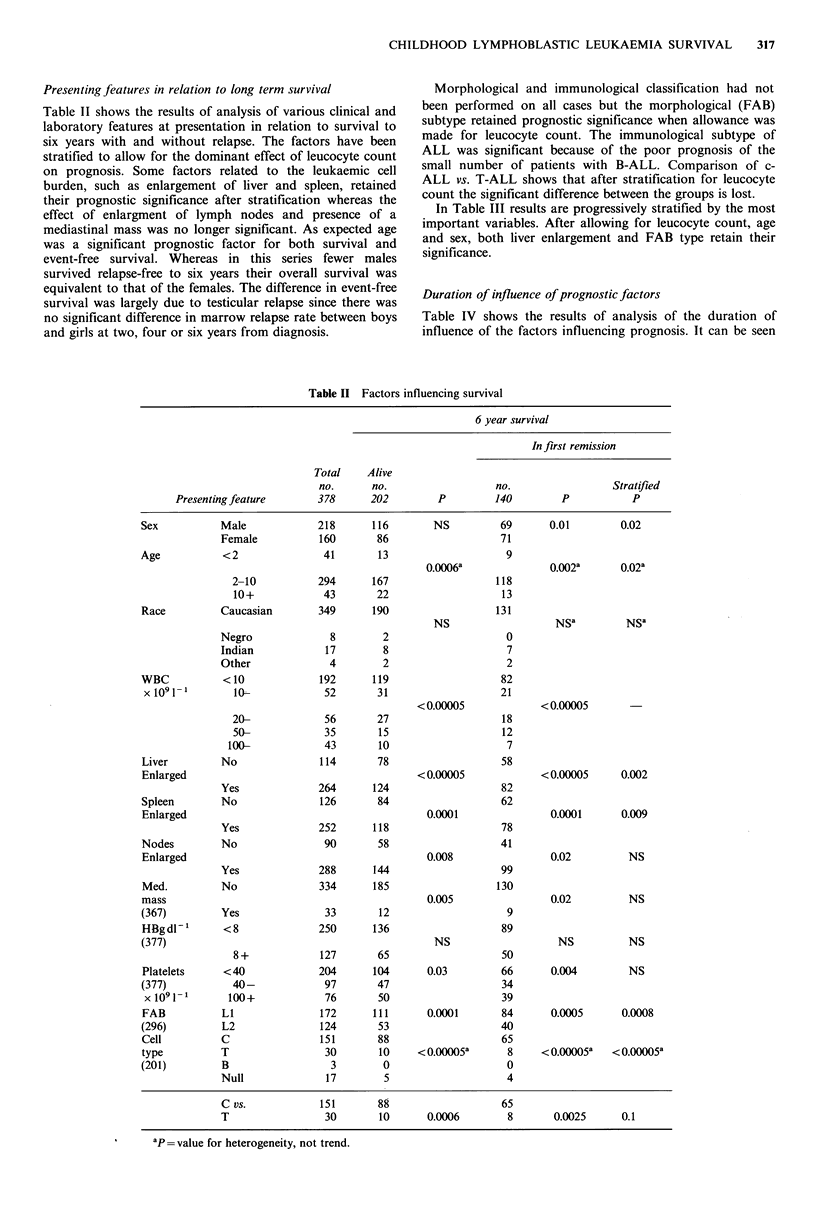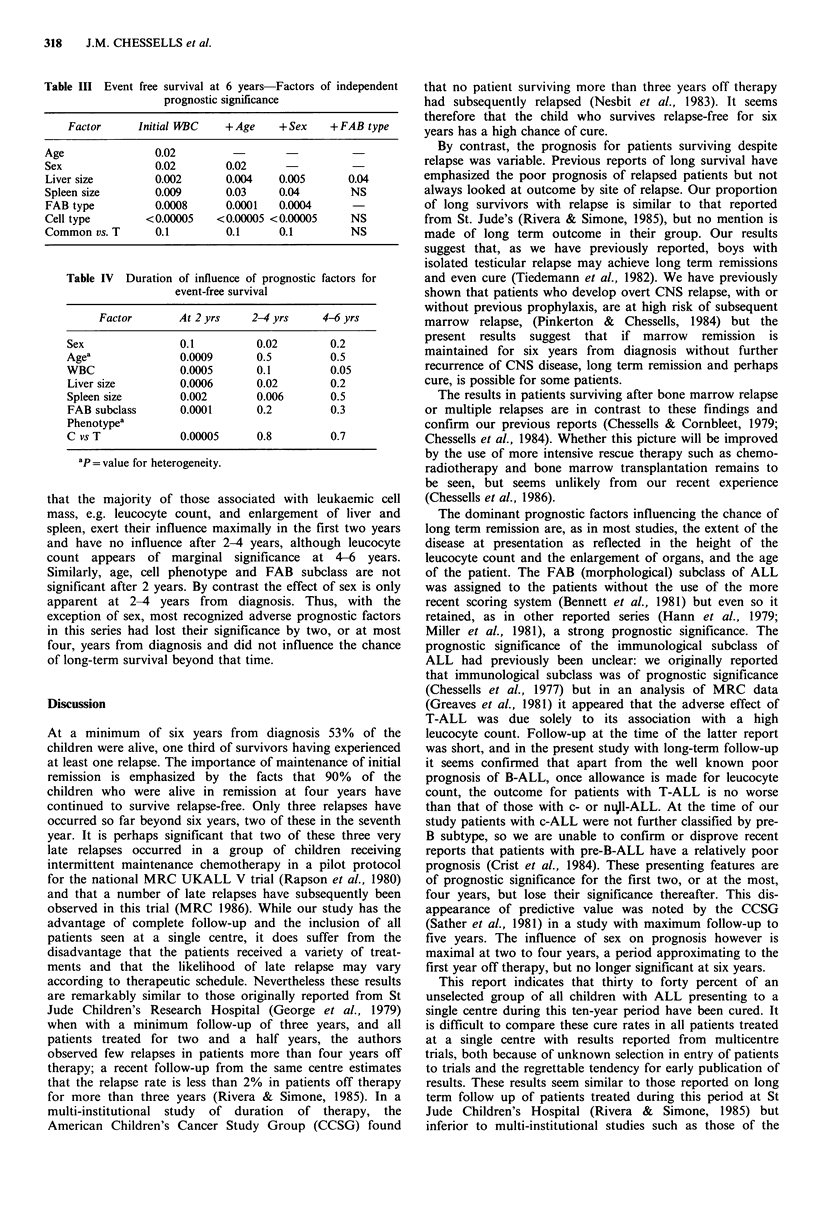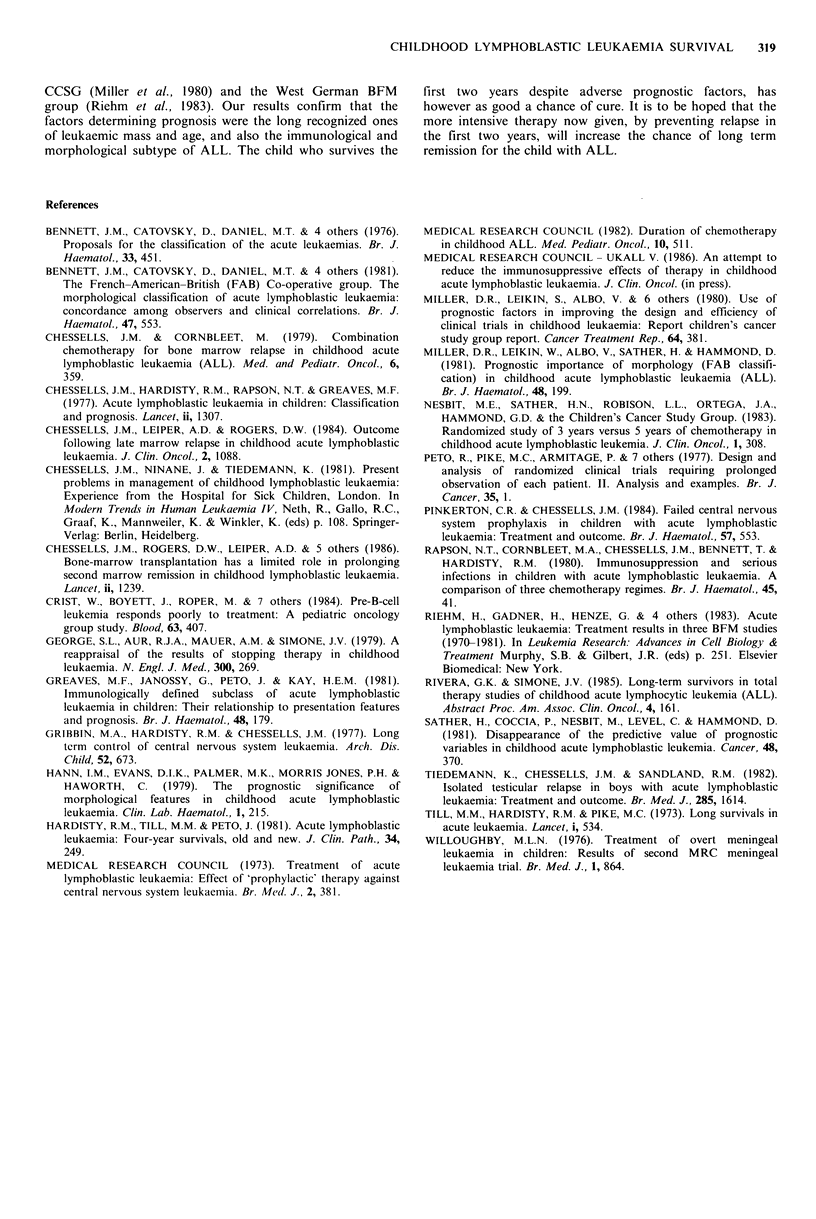# Long survival in childhood lymphoblastic leukaemia.

**DOI:** 10.1038/bjc.1987.62

**Published:** 1987-03

**Authors:** J. M. Chessells, R. M. Hardisty, S. Richards

## Abstract

Long term follow-up of 378 children with acute lymphoblastic leukaemia (ALL) treated at a single centre showed that at six years from diagnosis 202 (53%) were alive, of whom 140 (37%) remained in first remission. Only three children had a first relapse after six years. Children who survived six years despite a single extramedullary relapse in the testis or CNS were likely to remain in second remission but patients with previous marrow or with multiple relapses continued at risk for up to ten years from diagnosis. Presenting factors influencing event-free survival were: leucocyte count, age and sex. After allowing for these factors morphological (FAB) subtype and liver enlargement retained their prognostic significance. Immunological type of ALL was not of independent prognostic significance, except for the small number of patients with B-ALL. Most factors lost their significance after 2-4 years. It is concluded that patients alive 6 years from diagnosis without relapse or even with a single extramedullary relapse of ALL, have a high chance of prolonged survival and cure.


Br. J. Cancer (1987), 55, 315–319 © The Macmillan Press Ltd., 1987

### Long survival in childhood lymphoblastic leukaemia

J.M. Chessells[1], R.M. Hardisty[1] & S. Richards[2]

[1]*Department of Haematology and Oncology, Hospital for Sick Children, Great Ormond Street, London, WC1N 3JH and* [2]*Clinical Trials Service Unit, Oxford, OX2, 6BR, UK.*

**Summary** Long term follow-up of 378 children with acute lymphoblastic leukaemia (ALL) treated at a single centre showed that at six years from diagnosis 202 (53%) were alive, of whom 140 (37%) remained in first remission. Only three children had a first relapse after six years. Children who survived six years despite a single extramedullary relapse in the testis or CNS were likely to remain in second remission but patients with previous marrow or with multiple relapses continued at risk for up to ten years from diagnosis. Presenting factors influencing event-free survival were: leucocyte count, age and sex. After allowing for these factors morphological (FAB) subtype and liver enlargement retained their prognostic significance. Immunological type of ALL was not of independent prognostic significance, except for the small number of patients with B-ALL. Most factors lost their significance after 2–4 years.

It is concluded that patients alive 6 years from diagnosis without relapse or even with a single extramedullary relapse of ALL, have a high chance of prolonged survival and cure.

The definition of long survival in childhood lymphoblastic leukaemia (ALL) is an arbitrary one. Earlier reports of long survival have followed the fate of patients alive four years from diagnosis (Till *et al.*, 1973; Hardisty *et al.*, 1981) with emphasis on the importance of relapse-free survival and many trials of treatment are reported after about this duration of follow up. Since the introduction of 'modern' therapy most children with ALL have been treated for two or three years, and while most relapses occur either during treatment or within a year of stopping it, later relapses may occur (George *et al.*, 1979). We have therefore examined the outcome in all children referred to the Hospital for Sick Children since the introduction of modern therapy, who have been followed up for a minimum period of at least six years, in order to ascertain the risk and pattern of later relapses. We have also examined the outcome in children who were alive six years from diagnosis after one or more relapses, to determine which types of patient in this category had a chance of long term survival.

### Patients and methods

The patients comprise all children referred to the Hospital for Sick Children for treatment of ALL between 1970 and 1979. All were treated on protocols devised or being piloted for the Medical Research Council (MRC) Working Party on Childhood Leukaemia which have been described in detail elsewhere; (Chessells *et al.*, 1981). All patients received induction therapy with prednisolone, vincristine and at least one other drug and continuing multiple agent chemotherapy. Measures to prevent the development of overt leukaemic inflitration of the central nervous system (CNS prophylaxis) were used in all patients except those in UKALL I, (i.e. those referred in 1970 and 1971) and comprised cranial irradiation together with a course of intrathecal methotrexate injections and/or spinal irradiation. Many patients were involved in trials of optimal duration of chemotherapy, reported elsewhere (MRC, 1982), the longest duration being three years. Thus, at the time of analysis, all patients still in their first remission had been off treatment for at least three years.

Patients who had had a bone marrow relapse had received a variety of treatments, many of which have been described elsewhere (Chessells & Cornbleet, 1979; Chessells *et al.*, 1984). Management of extramedullary relapse evolved over the period under review. Patients in the MRC UKALL I (MRC 1973) trial did not receive CNS prophylaxis and a large number developed CNS relapse, many of whom were entered into the second MRC meningeal trial (Willoughby, 1976) which involved treatment of meningeal relapse with intrathecal methotrexate and a subsequent randomised comparison of cranial and craniospinal irradiation; patients relapsing later all received craniospinal irradiation as previously described (Gribbin *et al.*, 1977). The management of children relapsing despite CNS prophylaxis evolved to a standard policy of intrathecal chemotherapy, further systemic intensification and further, craniospinal, irradiation (Pinkerton & Chessells, 1984). Similarly the first few patients with localized testicular relapses did not receive standard chemotherapy and radiotherapy, but subsequently all patients were treated with bilateral testicular irradiation, systemic reinduction and intensification, intrathecal chemotherapy, and two years of further chemotherapy (Tiedemann *et al.*, 1982). All the patients have been followed up for a minimum of 6 years from diagnosis and no patients have been lost to follow-up.

### *Morphological and immunological analyses*

Morphological (FAB) classification of the leukaemia as originally described by Bennett *et al.* (1976), had been carried out at diagnosis on the majority of patients. The leukaemias had been classified immunologically by techniques then available (Greaves *et al.*, 1981) into c-ALL, T-ALL, B-ALL and null-ALL

### *Statistical analyses*

All statistical analyses were performed by the log-rank method (Peto *et al.*, 1977). After ascertainment of prognostic variables by unstratified analysis, results were stratified by the most important variables, leucocyte count and age, to determine which were of independent prognostic significance. In order to determine the duration of influence of prognostic variables, analyses were done for each of the periods, up to two years, two to four years and four to six years from diagnosis.

### Results

### *Long term disease-free survival after four and six years*

Three hundred and seventy-eight children with ALL were treated between 1970 and 1979 of whom 151 (40%) were

Correspondence: J.M. Chessells.
Received 14 August 1986; and in revised form 7 November 1986.

alive in first remission at four years. Since previous analyses had examined the outcome for patients from four years, we looked first at follow up of all patients in remission at four years and survival is shown for these 151 patients in Figure 1. It can be seen that the majority of relapses occurred in the fifth and sixth year from diagnosis. Thus, at six years, our time of minimum follow-up, 140 (37%) of all children and 93% of those in remission at four years, remained in remission; two relapses occurred in the seventh year (fourth year off treatment) and one in the eighth year. In all the cases of late relapse the morphological and cytochemical appearance of the leukaemic blast cells was consistent with ALL and, where immunological characterization had been performed, there was no evidence of alteration in phenotype; there was thus no reason to suppose that these relapses represented a new leukaemia.

**Figure 1** Disease-free survival for patients who were in first complete remission at 4 years. The graph shows follow-up from 4 years. Figures indicate numbers at risk. The standard error at 5 years is ±1.9%, at 7.5 years ±2.3% and at 10 and 12.5 years ±2.4%.

The 137 patients alive in first remission remain in general well and a detailed analysis of their health is in preparation. No second neoplasms have been observed among these six-year disease-free survivors, although in the whole group of 378 children there have been three. One child developed acute myeloid leukaemia two years from diagnosis and two children developed second neoplasms after bone marrow relapse, one Hodgkin's disease and one a cerebral astrocytoma. All three patients died within 6 years from diagnosis.

*Survival after relapse*

At six years from diagnosis there were 202 children alive of whom 62 had survived after one or more relapses. Twenty-one of these patients had relapsed on more than one occasion before six years, and, as shown in Figure 2, they continued thereafter at risk of further recurrence. Forty-one children had survived to six years from diagnosis having had a single relapse; Table I shows that these survivors comprise a minority of all those relapsing. Only 22 of the 134 children relapsing in the bone marrow, with or without concurrent relapse in the CNS or testis were alive and in second remission at six years and the risk of further relapse, as

**Figure 2** Follow up of patients who were alive at 6 years after one or more relapses. The curves indicate time to next relapse or death. There are two patients with combined marrow and CNS relapse included in the BM curve. The curve for multiple relapses indicates patients who had survived for 6 years having had more than one relapse.

**Table I** Six year survival after a single relapse

| *Site of relapse*[a] | *No. relapses* | *No. alive at 6 years in second remission* |
|---|---|---|
| Bone marrow ± other site | 134 | 22 |
| Central nervous system | | |
| Pre-prophylaxis (standard therapy) | 23 (9) | 4 (4) |
| Post-prophylaxis (standard therapy) | 26 (13) | 2 (2) |
| Testis (biopsy positive) | 18 (6) | 13 (6) |
| Total | 201 | 41 |

[a]Fifteen of 378 children did not achieve remission and 19 died in first remission. One patient developed acute myelogenous leukaemia, one relapsed in the eye and one in lymph nodes.

shown in Figure 2 continued after this time. Only 6 of the 49 patients experiencing CNS relapse as a first event survived in next remission to six years. Table I shows both the proportion of children relapsing in 1970 and 1971 before the routine use of 'prophylaxis' and those relapsing later, and also the proportion of children receiving what might in retrospect be considered best standard therapy with re-induction, intrathecal chemotherapy and craniospinal irradiation. Although the proportion surviving to six years was small thereafter none have subsequently relapsed. Eighteen boys (Table I) had an isolated testicular relapse as a first event and of these only one, a boy with B-ALL, relapsed during treatment. The others, as shown in the table all had a positive biopsy at two or three years of treatment or developed a relapse after stopping treatment. One of these 17 children received no further treatment, two received local irradiation alone and one received half the usual dose of irradiation (12 Gy instead of 24 Gy) to his other clinically uninvolved testis. Thus, 13 patients received standard treatment of whom twelve remain in remission six years and more from diagnosis. The failure on Figure 2 represents local recurrence in the boy receiving the low irradiation dose. Thus, it appears that if children with extramedullary disease survive in second remission for six years they may achieve long survival, that, as expected the outcome for the multiply relapsed patient is uncertain, and that patients remain at long term risk of recurrence for many years after bone marrow relapse.

*Presenting features in relation to long term survival*

Table II shows the results of analysis of various clinical and laboratory features at presentation in relation to survival to six years with and without relapse. The factors have been stratified to allow for the dominant effect of leucocyte count on prognosis. Some factors related to the leukaemic cell burden, such as enlargement of liver and spleen, retained their prognostic significance after stratification whereas the effect of enlargment of lymph nodes and presence of a mediastinal mass was no longer significant. As expected age was a significant prognostic factor for both survival and event-free survival. Whereas in this series fewer males survived relapse-free to six years their overall survival was equivalent to that of the females. The difference in event-free survival was largely due to testicular relapse since there was no significant difference in marrow relapse rate between boys and girls at two, four or six years from diagnosis.

Morphological and immunological classification had not been performed on all cases but the morphological (FAB) subtype retained prognostic significance when allowance was made for leucocyte count. The immunological subtype of ALL was significant because of the poor prognosis of the small number of patients with B-ALL. Comparison of c-ALL *vs.* T-ALL shows that after stratification for leucocyte count the significant difference between the groups is lost.

In Table III results are progressively stratified by the most important variables. After allowing for leucocyte count, age and sex, both liver enlargement and FAB type retain their significance.

*Duration of influence of prognostic factors*

Table IV shows the results of analysis of the duration of influence of the factors influencing prognosis. It can be seen

**Table II** Factors influencing survival

| | | | *6 year survival* | | | | |
|---|---|---|---|---|---|---|---|
| | | | | | *In first remission* | | |
| *Presenting feature* | | *Total no. 378* | *Alive no. 202* | *P* | *no. 140* | *P* | *Stratified P* |
| Sex | Male | 218 | 116 | NS | 69 | 0.01 | 0.02 |
| | Female | 160 | 86 | | 71 | | |
| Age | <2 | 41 | 13 | | 9 | | |
| | | | | 0.0006[a] | | 0.002[a] | 0.02[a] |
| | 2–10 | 294 | 167 | | 118 | | |
| | 10+ | 43 | 22 | | 13 | | |
| Race | Caucasian | 349 | 190 | | 131 | | |
| | | | | NS | | NS[a] | NS[a] |
| | Negro | 8 | 2 | | 0 | | |
| | Indian | 17 | 8 | | 7 | | |
| | Other | 4 | 2 | | 2 | | |
| WBC | <10 | 192 | 119 | | 82 | | |
| $\times 10^9 l^{-1}$ | 10– | 52 | 31 | | 21 | | |
| | | | | <0.00005 | | <0.00005 | — |
| | 20– | 56 | 27 | | 18 | | |
| | 50– | 35 | 15 | | 12 | | |
| | 100– | 43 | 10 | | 7 | | |
| Liver | No | 114 | 78 | | 58 | | |
| Enlarged | | | | <0.00005 | | <0.00005 | 0.002 |
| | Yes | 264 | 124 | | 82 | | |
| Spleen | No | 126 | 84 | | 62 | | |
| Enlarged | | | | 0.0001 | | 0.0001 | 0.009 |
| | Yes | 252 | 118 | | 78 | | |
| Nodes | No | 90 | 58 | | 41 | | |
| Enlarged | | | | 0.008 | | 0.02 | NS |
| | Yes | 288 | 144 | | 99 | | |
| Med. | No | 334 | 185 | | 130 | | |
| mass | | | | 0.005 | | 0.02 | NS |
| (367) | Yes | 33 | 12 | | 9 | | |
| HBg $dl^{-1}$ | <8 | 250 | 136 | | 89 | | |
| (377) | | | | NS | | NS | NS |
| | 8+ | 127 | 65 | | 50 | | |
| Platelets | <40 | 204 | 104 | 0.03 | 66 | 0.004 | NS |
| (377) | 40– | 97 | 47 | | 34 | | |
| $\times 10^9 l^{-1}$ | 100+ | 76 | 50 | | 39 | | |
| FAB | L1 | 172 | 111 | 0.0001 | 84 | 0.0005 | 0.0008 |
| (296) | L2 | 124 | 53 | | 40 | | |
| Cell | C | 151 | 88 | | 65 | | |
| type | T | 30 | 10 | <0.00005[a] | 8 | <0.00005[a] | <0.00005[a] |
| (201) | B | 3 | 0 | | 0 | | |
| | Null | 17 | 5 | | 4 | | |
| | C *vs.* | 151 | 88 | | 65 | | |
| | T | 30 | 10 | 0.0006 | 8 | 0.0025 | 0.1 |

[a]$P$ = value for heterogeneity, not trend.

**Table III** Event free survival at 6 years—Factors of independent prognostic significance

| *Factor* | *Initial WBC* | *+Age* | *+Sex* | *+FAB type* |
|---|---|---|---|---|
| Age | 0.02 | — | — | — |
| Sex | 0.02 | 0.02 | — | — |
| Liver size | 0.002 | 0.004 | 0.005 | 0.04 |
| Spleen size | 0.009 | 0.03 | 0.04 | NS |
| FAB type | 0.0008 | 0.0001 | 0.0004 | — |
| Cell type | <0.00005 | <0.00005 | <0.00005 | NS |
| Common *vs.* T | 0.1 | 0.1 | 0.1 | NS |

**Table IV** Duration of influence of prognostic factors for event-free survival

| *Factor* | *At 2 yrs* | *2–4 yrs* | *4–6 yrs* |
|---|---|---|---|
| Sex | 0.1 | 0.02 | 0.2 |
| Age[a] | 0.0009 | 0.5 | 0.5 |
| WBC | 0.0005 | 0.1 | 0.05 |
| Liver size | 0.0006 | 0.02 | 0.2 |
| Spleen size | 0.002 | 0.006 | 0.5 |
| FAB subclass | 0.0001 | 0.2 | 0.3 |
| Phenotype[a] | | | |
| C *vs* T | 0.00005 | 0.8 | 0.7 |

[a]$P$ = value for heterogeneity.

that the majority of those associated with leukaemic cell mass, e.g. leucocyte count, and enlargement of liver and spleen, exert their influence maximally in the first two years and have no influence after 2–4 years, although leucocyte count appears of marginal significance at 4–6 years. Similarly, age, cell phenotype and FAB subclass are not significant after 2 years. By contrast the effect of sex is only apparent at 2–4 years from diagnosis. Thus, with the exception of sex, most recognized adverse prognostic factors in this series had lost their significance by two, or at most four, years from diagnosis and did not influence the chance of long-term survival beyond that time.

### Discussion

At a minimum of six years from diagnosis 53% of the children were alive, one third of survivors having experienced at least one relapse. The importance of maintenance of initial remission is emphasized by the facts that 90% of the children who were alive in remission at four years have continued to survive relapse-free. Only three relapses have occurred so far beyond six years, two of these in the seventh year. It is perhaps significant that two of these three very late relapses occurred in a group of children receiving intermittent maintenance chemotherapy in a pilot protocol for the national MRC UKALL V trial (Rapson *et al.*, 1980) and that a number of late relapses have subsequently been observed in this trial (MRC 1986). While our study has the advantage of complete follow-up and the inclusion of all patients seen at a single centre, it does suffer from the disadvantage that the patients received a variety of treatments and that the likelihood of late relapse may vary according to therapeutic schedule. Nevertheless these results are remarkably similar to those originally reported from St Jude Children's Research Hospital (George *et al.*, 1979) when with a minimum follow-up of three years, and all patients treated for two and a half years, the authors observed few relapses in patients more than four years off therapy; a recent follow-up from the same centre estimates that the relapse rate is less than 2% in patients off therapy for more than three years (Rivera & Simone, 1985). In a multi-institutional study of duration of therapy, the American Children's Cancer Study Group (CCSG) found that no patient surviving more than three years off therapy had subsequently relapsed (Nesbit *et al.*, 1983). It seems therefore that the child who survives relapse-free for six years has a high chance of cure.

By contrast, the prognosis for patients surviving despite relapse was variable. Previous reports of long survival have emphasized the poor prognosis of relapsed patients but not always looked at outcome by site of relapse. Our proportion of long survivors with relapse is similar to that reported from St. Jude's (Rivera & Simone, 1985), but no mention is made of long term outcome in their group. Our results suggest that, as we have previously reported, boys with isolated testicular relapse may achieve long term remissions and even cure (Tiedemann *et al.*, 1982). We have previously shown that patients who develop overt CNS relapse, with or without previous prophylaxis, are at high risk of subsequent marrow relapse, (Pinkerton & Chessells, 1984) but the present results suggest that if marrow remission is maintained for six years from diagnosis without further recurrence of CNS disease, long term remission and perhaps cure, is possible for some patients.

The results in patients surviving after bone marrow relapse or multiple relapses are in contrast to these findings and confirm our previous reports (Chessells & Cornbleet, 1979; Chessells *et al.*, 1984). Whether this picture will be improved by the use of more intensive rescue therapy such as chemoradiotherapy and bone marrow transplantation remains to be seen, but seems unlikely from our recent experience (Chessells *et al.*, 1986).

The dominant prognostic factors influencing the chance of long term remission are, as in most studies, the extent of the disease at presentation as reflected in the height of the leucocyte count and the enlargement of organs, and the age of the patient. The FAB (morphological) subclass of ALL was assigned to the patients without the use of the more recent scoring system (Bennett *et al.*, 1981) but even so it retained, as in other reported series (Hann *et al.*, 1979; Miller *et al.*, 1981), a strong prognostic significance. The prognostic significance of the immunological subclass of ALL had previously been unclear: we originally reported that immunological subclass was of prognostic significance (Chessells *et al.*, 1977) but in an analysis of MRC data (Greaves *et al.*, 1981) it appeared that the adverse effect of T-ALL was due solely to its association with a high leucocyte count. Follow-up at the time of the latter report was short, and in the present study with long-term follow-up it seems confirmed that apart from the well known poor prognosis of B-ALL, once allowance is made for leucocyte count, the outcome for patients with T-ALL is no worse than that of those with c- or null-ALL. At the time of our study patients with c-ALL were not further classified by pre-B subtype, so we are unable to confirm or disprove recent reports that patients with pre-B-ALL have a relatively poor prognosis (Crist *et al.*, 1984). These presenting features are of prognostic significance for the first two, or at the most, four years, but lose their significance thereafter. This disappearance of predictive value was noted by the CCSG (Sather *et al.*, 1981) in a study with maximum follow-up to five years. The influence of sex on prognosis however is maximal at two to four years, a period approximating to the first year off therapy, but no longer significant at six years.

This report indicates that thirty to forty percent of an unselected group of all children with ALL presenting to a single centre during this ten-year period have been cured. It is difficult to compare these cure rates in all patients treated at a single centre with results reported from multicentre trials, both because of unknown selection in entry of patients to trials and the regrettable tendency for early publication of results. These results seem similar to those reported on long term follow up of patients treated during this period at St Jude Children's Hospital (Rivera & Simone, 1985) but inferior to multi-institutional studies such as those of the

CCSG (Miller *et al.*, 1980) and the West German BFM group (Riehm *et al.*, 1983). Our results confirm that the factors determining prognosis were the long recognized ones of leukaemic mass and age, and also the immunological and morphological subtype of ALL. The child who survives the first two years despite adverse prognostic factors, has however as good a chance of cure. It is to be hoped that the more intensive therapy now given, by preventing relapse in the first two years, will increase the chance of long term remission for the child with ALL.

### References

BENNETT, J.M., CATOVSKY, D., DANIEL, M.T. & 4 others (1976). Proposals for the classification of the acute leukaemias. *Br. J. Haematol.*, **33**, 451.

BENNETT, J.M., CATOVSKY, D., DANIEL, M.T. & 4 others (1981). The French–American–British (FAB) Co-operative group. The morphological classification of acute lymphoblastic leukaemia: concordance among observers and clinical correlations. *Br. J. Haematol.*, **47**, 553.

CHESSELLS, J.M. & CORNBLEET, M. (1979). Combination chemotherapy for bone marrow relapse in childhood acute lymphoblastic leukaemia (ALL). *Med. and Pediatr. Oncol.*, **6**, 359.

CHESSELLS, J.M., HARDISTY, R.M., RAPSON, N.T. & GREAVES, M.F. (1977). Acute lymphoblastic leukaemia in children: Classification and prognosis. *Lancet*, **ii**, 1307.

CHESSELLS, J.M., LEIPER, A.D. & ROGERS, D.W. (1984). Outcome following late marrow relapse in childhood acute lymphoblastic leukaemia. *J. Clin. Oncol.*, **2**, 1088.

CHESSELLS, J.M., NINANE, J. & TIEDEMANN, K. (1981). Present problems in management of childhood lymphoblastic leukaemia: Experience from the Hospital for Sick Children, London. In *Modern Trends in Human Leukaemia IV*, Neth, R., Gallo, R.C., Graaf, K., Mannweiler, K. & Winkler, K. (eds) p. 108. Springer-Verlag: Berlin, Heidelberg.

CHESSELLS, J.M., ROGERS, D.W., LEIPER, A.D. & 5 others (1986). Bone-marrow transplantation has a limited role in prolonging second marrow remission in childhood lymphoblastic leukaemia. *Lancet*, **ii**, 1239.

CRIST, W., BOYETT, J., ROPER, M. & 7 others (1984). Pre-B-cell leukemia responds poorly to treatment: A pediatric oncology group study. *Blood*, **63**, 407.

GEORGE, S.L., AUR, R.J.A., MAUER, A.M. & SIMONE, J.V. (1979). A reappraisal of the results of stopping therapy in childhood leukaemia. *N. Engl. J. Med.*, **300**, 269.

GREAVES, M.F., JANOSSY, G., PETO, J. & KAY, H.E.M. (1981). Immunologically defined subclass of acute lymphoblastic leukaemia in children: Their relationship to presentation features and prognosis. *Br. J. Haematol.*, **48**, 179.

GRIBBIN, M.A., HARDISTY, R.M. & CHESSELLS, J.M. (1977). Long term control of central nervous system leukaemia. *Arch. Dis. Child*, **52**, 673.

HANN, I.M., EVANS, D.I.K., PALMER, M.K., MORRIS JONES, P.H. & HAWORTH, C. (1979). The prognostic significance of morphological features in childhood acute lymphoblastic leukaemia. *Clin. Lab. Haematol.*, **1**, 215.

HARDISTY, R.M., TILL, M.M. & PETO, J. (1981). Acute lymphoblastic leukaemia: Four-year survivals, old and new. *J. Clin. Path.*, **34**, 249.

MEDICAL RESEARCH COUNCIL (1973). Treatment of acute lymphoblastic leukaemia: Effect of 'prophylactic' therapy against central nervous system leukaemia. *Br. Med. J.*, **2**, 381.

MEDICAL RESEARCH COUNCIL (1982). Duration of chemotherapy in childhood ALL. *Med. Pediatr. Oncol.*, **10**, 511.

MEDICAL RESEARCH COUNCIL – UKALL V. (1986). An attempt to reduce the immunosuppressive effects of therapy in childhood acute lymphoblastic leukaemia. *J. Clin. Oncol.* (in press).

MILLER, D.R., LEIKIN, S., ALBO, V. & 6 others (1980). Use of prognostic factors in improving the design and efficiency of clinical trials in childhood leukaemia: Report children's cancer study group report. *Cancer Treatment Rep.*, **64**, 381.

MILLER, D.R., LEIKIN, W., ALBO, V., SATHER, H. & HAMMOND, D. (1981). Prognostic importance of morphology (FAB classification) in childhood acute lymphoblastic leukaemia (ALL). *Br. J. Haematol.*, **48**, 199.

NESBIT, M.E., SATHER, H.N., ROBISON, L.L., ORTEGA, J.A., HAMMOND, G.D. & the Children's Cancer Study Group. (1983). Randomized study of 3 years versus 5 years of chemotherapy in childhood acute lymphoblastic leukemia. *J. Clin. Oncol.*, **1**, 308.

PETO, R., PIKE, M.C., ARMITAGE, P. & 7 others (1977). Design and analysis of randomized clinical trials requiring prolonged observation of each patient. II. Analysis and examples. *Br. J. Cancer*, **35**, 1.

PINKERTON, C.R. & CHESSELLS, J.M. (1984). Failed central nervous system prophylaxis in children with acute lymphoblastic leukaemia: Treatment and outcome. *Br. J. Haematol.*, **57**, 553.

RAPSON, N.T., CORNBLEET, M.A., CHESSELLS, J.M., BENNETT, T. & HARDISTY, R.M. (1980). Immunosuppression and serious infections in children with acute lymphoblastic leukaemia. A comparison of three chemotherapy regimes. *Br. J. Haematol.*, **45**, 41.

RIEHM, H., GADNER, H., HENZE, G. & 4 others (1983). Acute lymphoblastic leukaemia: Treatment results in three BFM studies (1970–1981). In *Leukemia Research: Advances in Cell Biology & Treatment* Murphy, S.B. & Gilbert, J.R. (eds) p. 251. Elsevier Biomedical: New York.

RIVERA, G.K. & SIMONE, J.V. (1985). Long-term survivors in total therapy studies of childhood acute lymphocytic leukemia (ALL). *Abstract Proc. Am. Assoc. Clin. Oncol.*, **4**, 161.

SATHER, H., COCCIA, P., NESBIT, M., LEVEL, C. & HAMMOND, D. (1981). Disappearance of the predictive value of prognostic variables in childhood acute lymphoblastic leukemia. *Cancer*, **48**, 370.

TIEDEMANN, K., CHESSELLS, J.M. & SANDLAND, R.M. (1982). Isolated testicular relapse in boys with acute lymphoblastic leukaemia: Treatment and outcome. *Br. Med. J.*, **285**, 1614.

TILL, M.M., HARDISTY, R.M. & PIKE, M.C. (1973). Long survivals in acute leukaemia. *Lancet*, **i**, 534.

WILLOUGHBY, M.L.N. (1976). Treatment of overt meningeal leukaemia in children: Results of second MRC meningeal leukaemia trial. *Br. Med. J.*, **1**, 864.